# Characterization of a Recombinant Laccase B from *Trametes hirsuta* MX2 and Its Application for Decolorization of Dyes

**DOI:** 10.3390/molecules27051581

**Published:** 2022-02-27

**Authors:** Yitong Jia, Qianqian Huang, Lanlan Zhu, Chengyuan Pan

**Affiliations:** 1 The Key Laboratory for Quality Improvement of Agricultural Products of Zhejiang Province, College of Advanced Agricultural Sciences, Zhejiang A&F University, Hangzhou 311300, China; 2019601061002@stu.zafu.edu.cn (Y.J.); 2017701502006@stu.zafu.edu.cn (Q.H.); 2 Science and Technology Service Center of Lin’an, Hangzhou 311300, China

**Keywords:** laccase, *Trametes hirsuta*, dye, decolorization, characterization, heterologous expression

## Abstract

*Trametes hirsuta* is able to secrete laccase isoenzymes including constitutive and inducible forms, and has potential application for bioremediation of environmental pollutants. Here, an inducible group B laccase from *T. hirsuta* MX2 was heterologously expressed in *Pichia pastoris*, and its yield reached 2.59 U/mL after 5 days of methanol inducing culture. The optimal pH and temperature of recombinant laccase (rLac1) to 2,2′-azino-bis-(3-ethylbenzothiazoline-6-sulfonic acid) (ABTS) were 2.5 and 60 °C, respectively. Metal ions showed different effect on rLac1 which Mg^2+^, Cu^2+^, and K^+^ increased enzyme activity as their concentration increased, whereas Zn^2+^, Na^+^, and Fe^2+^ inhibited enzyme activity as their concentration increased. rLac1 showed good tolerance to organic solvents, and more than 42% of its initial activity remained in 10% organic solvents. Additionally, rLac1 exhibited a more efficient decolorization ability for remazol brilliant blue R (RBBR) than for acid red 1 (AR1), crystal violet (CV), and neutral red (NR). Molecular docking results showed RBBR has a stronger binding affinity with laccase than other dyes by interacting with substrate binding cavity of enzyme. The results indicated rLac1 may be a potential candidate for dye removal from textile wastewater.

## 1. Introduction

Synthetic dyes including anthraquinone, azo, heterocycle, and triphenylmethane are widely used in textile, tannery and printing, cosmetic, paper, and pharmaceutical industries [1]. It is estimated that more than 100,000 synthetic dyes are available globally, with annual production of over 1 million tons [2]. However, a large amount of dyes is released into wastewater in their industrial applications. For instance, about 20–50% of dyes are discharged into effluent during textile processing [3]. The recalcitrant chemical structure of some dyes and their intense coloration in water pose a serious threat to aquatic organisms [4,5]. The removal of dyes by adsorption, coagulation, ozonation, and chemical degradation is often high-cost and can generate hazardous by-products [6]. Biodegradation of synthetic dyes by enzymes has attracted increasing attention in recent years because its advantage of high efficiency and eco-friendly procedures in the treatment of dye effluent [7,8,9].

Laccase is a group of copper-containing oxidases that can catalyze the oxidation of phenolic and non-phenolic compounds, and is widely distributed in plants, fungi, bacteria, and insects [10]. In laccase-mediated catalytic reaction, water is the sole by-product produced by the transfer of electrons from hydrogen donating substrate to molecular oxygen. The extensive substrates and unique catalytic mechanism of laccase make it application in agricultural, industrial, medicinal, and environmental areas [11]. In recent years, more and more laccases have been proved to be the enzymes with great potential in the degradation of synthetic dyes [9,12,13]. The azonaphthol Orange 2 can be degraded by laccase from *Trametes versicolor*, with 72.8% decolorization [12]. Sun et al. observed that *Gymnopusluxurians* laccase exhibited efficient decolorization capability for 11 synthetic dyes with the help of the mediator of acetosyringone or syringaldehyde [14]. Among laccases derived from different organisms, fungal laccase has been considered as an ideal source of dye decolorization due to its high-redox potential [15].

*Trametes hirsuta* is a basidiomycete fungus that can secrete a variety of laccase isoenzymes [8,16,17]. Laccases obtained from different strains of *T. hirsuta* showed dissimilar decolorization ability to various types of dyes. For example, the crude laccase from *T. hirsuta* BT 2566 could decolorize more than 50% of the six azo dyes [18], and the laccase of *T. hirsuta* Bm-2 displayed 97% decolorization to indigo carmine using natural mediators of phenol extracts [19]. RBBR and indigosol dye could be successfully degraded by laccase from *T. hirsuta* EDN 082 immobilized on light expanded clay aggregate [20,21]. It was further discovered that the laccase isoenzymes are encoded by multigene families, which contain seven functional genes and one non-processed pseudogene in *T. hirsuta* 072 [22]. However, not all laccase genes are constitutively expressed in the fungal transcriptomes and most of them need to be induced to expression by phenolic substances [22]. Savinova et al. classified the laccase isoenzymes from the fungi of the Polyporales order into A–H according to the homology of their encoding genes [23]. Group A laccases, as the constitutive isoenzymes (major laccases), are always secreted in the fungal secretomes regardless of culture conditions and have been sufficiently studied [24,25,26]. Group B-H laccases are the inducible isoenzymes (minor laccases) whose gene transcription is mosaic depending on both cultivation stages and growth conditions [22,23]. To the best of our knowledge, only two group B laccases have been isolated and characterized from the original fungi. One was induced from *Trametesvillosa* by 2,5-xylidine, and its optimal pH was between 5 and 5.5 with syringaldazine as a substrate [27]. Another one was produced from *T. hirsuta* by growing in wood-based medium, and had an optimal pH of 2.5 to ABTS [8]. The limited data do not allow us to make definite inferences about group B laccase properties.

Heterologous expression is an effective method to produce inducible laccase isoenzymeswhich are expressed at low levels or not at all in the original fungi. Three of the six minor laccases of *T. hirsuta* 072 were successfully obtained by expressing them in *Penicillium canescens* [28]. In addition, heterologous expression may also improve recombinant enzyme yields and may allow the production of laccases with desirable characteristics. A yield of *Moniliophthoraroreri* laccase up to 1.05 g/L was detected when it was expressed in *Pichia pastoris* [29]. Compared to the native laccases, the recombinant laccases expressed heterologous host of *P*. *pastoris* showed more excellent properties including thermal stability, salt tolerance, specific activity, and alkaline resistance [30,31].

Previously, a laccase (Lac1) was purified and characterized from *T*. *hirsuta* MX2, and was identified as an inducible group B laccase by comparing its encoding gene with other fungal laccase gene sequences. Lac1 efficiently decolorizes synthetic dyes in the present of ABTS, but its yield from the original strain is relatively low to meet the industrial demand [8]. In this study, in order to increase the yield of Lac1, it was heterologously expressed in *P. pastoris*. The characteristics and dyes decolorization capability of recombinant Lac1 (rLac1) were investigated. The mechanism of dye decolorization by rLac1 was also elaborated employing molecular docking.

## 2. Results

### 2.1. Screening and Expression of Laccase Recombinants

**Figure 1:**
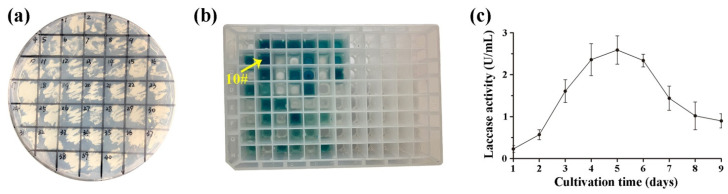
Screening of *P*. *pastoris* recombinants and laccase activity. (**a**) Growing of recombinants on a histidine-deficient MD agar plate. (**b**) Screening of recombinants with laccase activity in BMMY medium containing ABTS. (**c**) The activity of recombinant laccase in BMMY liquid medium. The values were mean ± SD.

A 1500 bp nucleotide sequence of *Lac1* without native signal peptide was amplified from *T. hirsuta* MX2 by using specific primers. The *Lac1* was ligated into pPIC9K to generate expression vector pPIC9K-*Lac1*, which was then integrated into the *P. pastoris* genome by electroporation. In MD screening medium, forty yeast recombinants were randomly selected for detection of laccase activity in a 96-well plate (Figure 1a). The positive recombinants with laccase activity were verified by adding ABTS into BMMY for color reaction, and a colony showing dark green (numbered of 10) was used for subsequent recombinant laccase production (Figure 1b). The colony was inoculated into 1 L BMMY medium and cultured under methanol induction. Laccase activity was detected in the culture liquid, and reached the highest value of 2.59 U/mL after 5 days of culture (Figure 1c).

### 2.2. Purification of Recombinant Laccase

**Figure 2:**
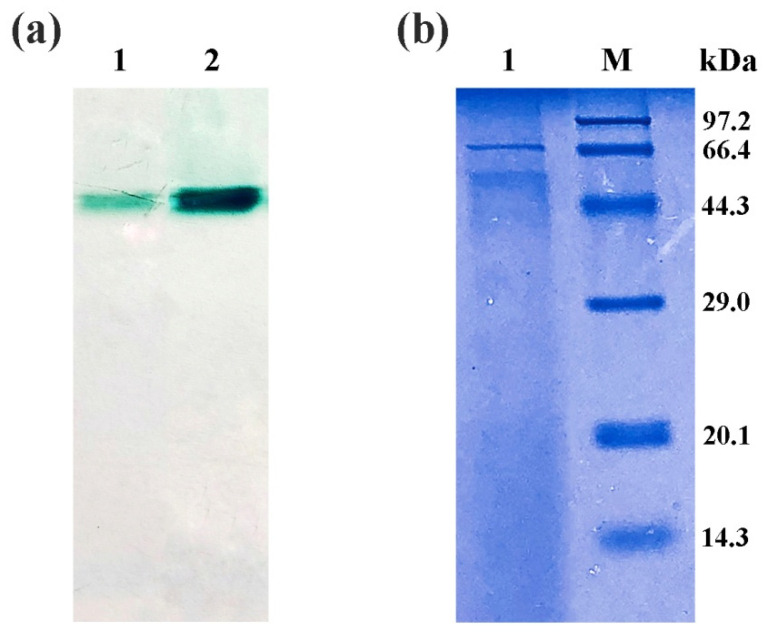
Electrophoresis of purified rLac1. (**a**) Zymogram analysis of rLac1 using ABTS as substrate, line 1 is rLac1 in the culture supernatant, and line 2 is purified rLac1. (**b**) Molecular weight analysis of rLac1 by SDS-PAGE, line 1 is purified rLac1, and line M is the protein marker.

Supernatant of the colony 10 cultured for 5 days in BMMY was used to purify rLac1. Purification steps included ultrafiltration, ammonium sulfate precipitation, and anion exchange chromatography. Laccase activity and protein concentration were determined for each purified product. Total activity was reduced from 2235 U in the culture supernatant to 129 U after purification, however specific activity was correspondingly increased from 19.1 to 92.1 U/mg (Table 1). It can be seen from the zymogram result in Figure 2a that there was only one band of rLac1 in both the culture supernatant and purified fraction. The deeper green band in the purified fraction than in the culture supernatant also indicated that rLac1 had been enriched after purification (Figure 2a). SDS-PAGE analysis showed that the molecular weight of rLac1 was about 63 kDa (Figure 2b).

### 2.3. Characterization of Recombinant Laccase

**Figure 3:**
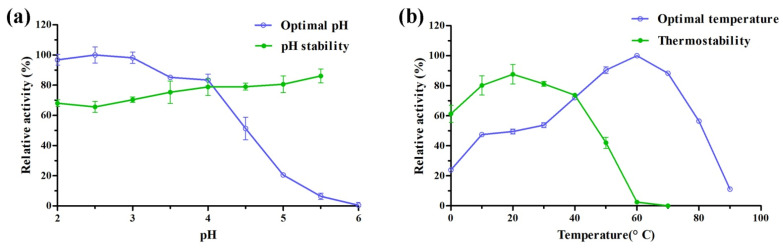
Effects of pH and temperature on the activity and stability of rLac1. (**a**) Optimal pH and stability of rLac1, the pH stability was showed as the percentage of residual activity after incubation at different pH values for 72 h. (**b**) Optimal temperature and stability of rLac1, the thermostability was showed as the percentage of residual activity after incubation at different temperatures for 30 min. The values were mean ± SD.

The purified rLac1 showed maximal oxidation activity to ABTS at pH 2.5 (Figure 3a). The activity declined slowly at pH 2.5–4.0, but decreased rapidly at pH above 4.0. rLac1 completely inactivated at pH ≥ 6.0. pH stability gradually increased when increasing reaction pH above 2.5. The residue activity of rLac1 remained between 65.6–86.2% after incubation at pH 2.0–5.5 for 72 h. rLac1displayed maximal activity at 60 °C, and the activity decreased rapidly when temperature was increased to 80 °C and higher (Figure 3b). Poor thermostability was observed from the results in Figure 3b when rLac1 was inoculated at temperature above 50 °C. The residue activity was close to zero after incubated at 60 °C for 30 min.

*K*_m_ and *k*_cat_ values of rLac1 were determined using ABTS and DMP as substrates. The results in Table 2 showed that ABTS was more easily bound by rLac1 than DMP with the *K*_m_ value of 28.4 µM for ABTS and *K*_m_ of 394.1 µM for DMP. rLac1 exhibited a higher catalytic turnover rate for ABTS (*k*_cat_ = 343.2 s^−1^) than DMP (*k*_cat_ = 141.7 s^−1^).

**Figure 4:**
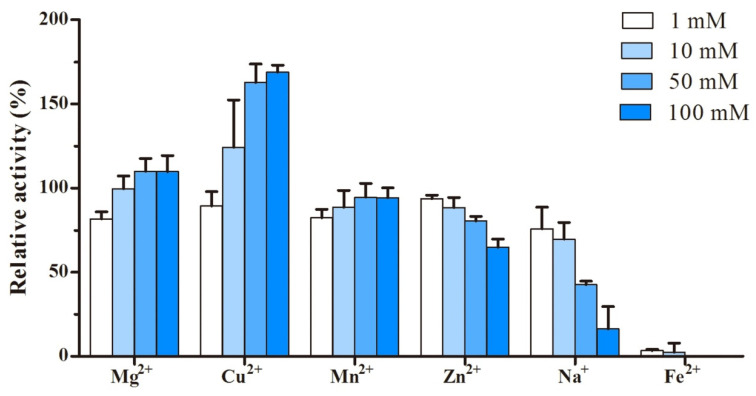
Effect of metal ions on the activity of rLac1. Activity was measured at 30 °C in citrate phosphate buffer (100 mM, pH 5.0). The values were mean ± SD.

The effect of metal ions on rLac1 activity was dose-dependent (Figure 4). Metal ions such as Mg^2+^, Cu^2+^, and K^+^ enhanced rLac1 activity with their concentration increased, especially Cu^2+^, which enhanced 68.9% of activity in the concentration of 100 mM. Conversely, the activities were inhibited by Zn^2+^ and Na^+^ with their concentration increased. rLac1 activity was almost completely inhibited by Fe^2+^, and only 3.2% of residual activity was determined in present of Fe^2+^ at 1 mM concentration.

The effects of organic solvents of acetone, acetonitrile, dimethylsulfoxide, ethanol, methanol, and isopropanol on rLac1 activity were investigated. rLac1 displayed some certain degrees of tolerance to six organic solvents. In presence of 5% and 10% organic solvents, the relative activities of rLac1 were more than 64% and 42%, respectively (Table 3). rLac1 was significantly inhibited by organic solvents at a concentration of 50%.

### 2.4. Dye Decolorization

The decolorization capability of rLac1 on anthraquinone (RBBR), azo (AR1), triphenylmethane (CV), and heterocycle (NR) was investigated. rLac1 exhibited a strong decolorization ability for RBBR with 92.57% of decolorization rate after 3 h reaction in the absence of ABTS, whereas the decolorization to AR1, CV, and NR was weaker and decolorization rates were 15.3%, 14.2%, and 12.3%, respectively (Table 4). When ABTS was added to the reaction, the decolorization rates of rLac1 to RBBR, AR1, CV, and NR were increased to 99.2%, 67.1%, 38.9%, and 52.3%, respectively.

### 2.5. Molecular Docking

**Figure 5:**
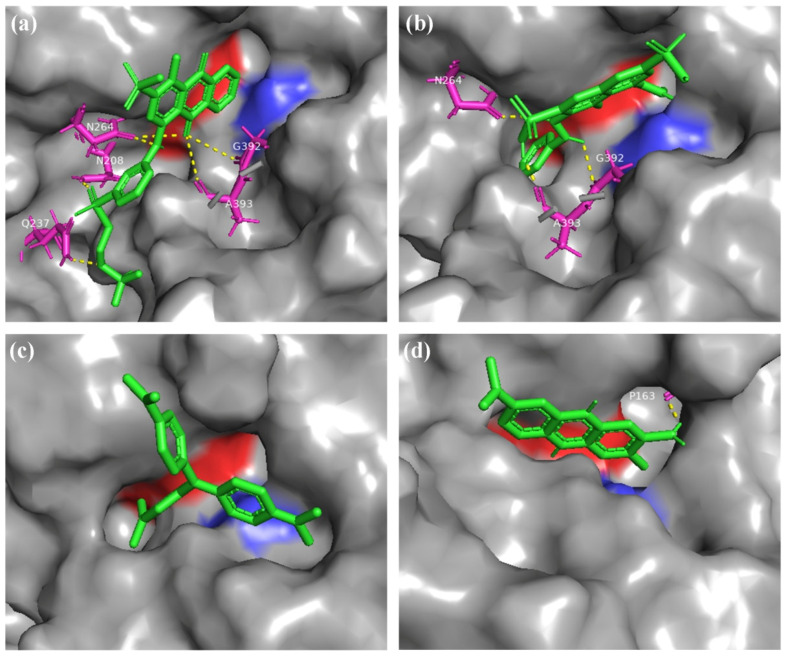
Molecular docking results of laccase with dye molecules of RBBR (**a**), AR1 (**b**), CV (**c**), or NR (**d**). The green and pink models represent dye molecules and their docking amino acids, respectively. The yellow dashes indicate H-bonds. The blue and red shadows represent conserved histidine (H_458_) and acidic aspartic acid (D_206_) in the substrate binding cavity of laccase, respectively.

Here, molecular docking was used to explore the interaction mechanism between laccase protein and dye molecules. All four dyes can bind to the SBC which located in the Cu T1 site of laccase (Figure 5). RBBR interacts with amino acid residues N208, Q237, N264, G392, and A393 on laccase via H-bond. N264, G392, and A393 were found as active site residues for laccase to interact with AR1. NR was observed to interact with laccase through P163, while no amino acid residue involved in the binding mechanism between CV and laccase. The binding energies of laccase with RBBR, AR1, CV, and NR were −6.8 kcal/mol, −6.4 kcal/mol, −6.5 kcal/mol, and −5.1 kcal/mol, respectively.

## 3. Discussions

Fungal laccases are promising enzymes for decolorization and detoxification of dyes, but the low production levels in original fungi limit their industrial applications. The expression of laccases in heterologous hosts, such as bacteria, filamentous fungi, and yeast is an effective method to increase their production [32]. Among those hosts, *P. pastoris* has received more attention because of its easy to manipulation and high protein secretion capacity [29,33,34], and has successfully expressed so far over 40 fungal laccases [35]. *T*. *hirsuta* is one of the fungi that intensively used to produce laccase. Two types of laccase, constitutive and inducible forms, can be secreted from the fungus [17]. LacA, as a constitutive laccase, has been expressed in filamentous fungus of *Aspergillus nidulans*, and the highest laccase activity of 2.0 U/mL was observed in the culture liquid [36]. Three inducible laccases of LacC, LacD, and LacF have been also expressed in *P. canescens*, and the activities of recombinant laccases were more than 100 U/mL in the culture broth [26]. In this study, Lac1, as an inducible laccase of group B from *T. hirsuta* MX2 [8], was firstly expressed in *P. pastoris*. A yeast recombinant with high laccase activity was screened, and its activity was five times higher than that of the original strain [8].

The purified recombinant laccase of rLac1 was used to compare with other group B laccases of *Trametes* in properties. As showed in Figure 2, rLac1 exhibited a slightly higher molecular weight than native Lac1 of 61.4 kDa reported previously [8]. Higher molecular weight of heterologous-expressed laccases has also been observed in other reports [30,35], which may be associated with higher glycosylation levels of recombinant laccases.

Generally, inducible laccases such as groups B-H are characterized with neutral optimal pH [27]. Koschorreck et al. found that the optimal pH of a recombinant group B laccase from *T. versicolor* was determined in 1.9 [37]. Similarly, optimal pH of rLac1 in acidic regions was displayed in this study. Although the optimal temperature of group B laccase could reach to 75 °C, it is generally not stable at high temperatures [27,37]. Previous study found that native Lac1 of *T. hirsuta* MX2 has a half-life of 0.88 h at 60 °C [8]. Here, rLac1 showed a lower thermostability than Lac1, and was almost inactive after incubated at 60 °C for 30 min (Figure 3).

ABTS is generally more suitable for use as a substrate for fungal laccase than DMP. This study also found that the affinity and catalytic efficiency of rLac1 to ABTS were better than that to DMP (Table 2). Compared with native Lac1, the *k*_cat_ values of rLac1 to ABTS and DMP were increased, which could be found in the study of Xu et al. [31].

Metal ions and organic solvents often occur in textile dye wastewater. If laccase is used to remove dyes from wastewater, it is necessary to evaluate the effect of these factors on laccase activity. In this study, rLac1 showed a strong tolerance to metal ions of Mg^2+^, Mn^2+^, and Zn^2+^, and its activity was still retained over 60% in the presence of these ions at 100 mM, while Fe^2+^ inhibited enzyme activity (Figure 4). Cu^2+^ is considered to be an inducer of laccase activity, but the activity promotion often occurs at low concentrations [34,38,39]. However, the activity of rLac1 was obviously improved by Cu^2+^ at high concentration (100 mM). In addition, Vasina et al. reported that *lacB*, encoding gene of group B laccase, was also expressed at a higher level induced by copper than other groups of laccase genes [40]. Therefore, it may be believed that Cu^2+^ is helpful to the improvement of the activity of group B laccase and its production in *Trametes*. Fungal laccase is usually highly sensitive to Fe^2+^, probably due to the competitive inhibition of Fe^2+^ in laccase electron transport [34,39,41]. The concentration of organic solvent seems to be a critical factor affecting laccase activity. At low concentration, organic solvents have little effect on laccase activity, and even promote it. For example, laccase activities from *Lentinula edodes* and *Kurthiahuakuii* were enhanced in the presence of 10–15% of methanol and ethanol [42,43]. However, most fungal laccases will become inactive in organic solvents above 10% [29,44]. Although no enhancement in rLac1 activity by solvents was observed in this study, rLac1 could tolerate organic solvents to some extent, and retained over 42% of the original activity in 10% organic solvents. The loss of laccase activity in the present of organic solvents may be caused by the unfolding of laccase protein or the competing binding of organic solvent to the active center of laccase [42,44].

*T. hirsuta* is considered to be a promising fungus in dye decolorization by employing its own secreted laccase isoenzymes. These isoenzymes perform different functions during dye decolorization, and inducible laccases generally exhibit better decolorization ability than constitutive laccases [8,28]. rLac1, as an inducible laccase, was found to have excellent decolorization of RBBR, and its decolorization rate is more than 90% regardless of the presence or absence of ABTS (Table 4). In comparison, the decolorization rate of laccase from *T. pubescens* and *Fusarium oxysporum* to RBBR is much lower in the absence of mediator [34,41]. ABTS is the first synthetic mediator for laccase, and has been shown to enhance the decolorization ability of laccase. This enhancement may be resulted from the involvement of ABTS in electron transfer during laccase-induced dye decolorization [45]. Similarly, the decolorization rates of AR1, CV, and NR by rLac1 in the presence of ABTS are significantly higher than that without ABTS in this study, indicating that the decolorization of these dyes by rLac1 requires the participation of mediator. In addition, a comparative analysis of recombinant rLac1 and native Lac1 to dye decolorization was conducted. The results showed the decolorization rates of rLac1 to RBBR, AR1, and NR were higher than that of Lac1 (Table 4) [8]. Similar observations were found in the recombinant laccase of *Coprinopsis cinerea*, in which the decolorization ability of recombinant laccase was improved compared with that of native laccase [31]. Although the mechanism of improving decolorization ability of recombinant laccases is not clear so far, they tend to have different glycosylation patterns and levels than the native laccases [35,46]. The glycosylation of recombinant laccases not only affects their physicochemical properties, but also may help to improve the decolorization ability of laccases [30].

The active site of laccase occurs around four copper ions, where T1 Cu is the site of substrate binding and oxidation. Dyes interact with amino acid residues around T1 Cu mainly by hydrogen bonding, and binding to laccase protein [47]. In molecular docking test, the binding site between dye and laccase was set in T1 Cu SBC, in which contains a highly conserved histidine and acidic aspartic acid (Figure 5) [48]. The results showed that RBBR displayed more hydrogen bonds and higher binding energy with laccase than other tested dyes, which may account for the higher decolorization rate of laccase to RBBR.

## 4. Materials and Methods

### 4.1. Strains, Vector, and Culture Media

*T. hirsuta* MX2 was isolated from decayed wood with high laccase activity, and was used as the source strain of laccase gene in this study [8]. pMD 19-T vector (TaKaRa, Kusatsu, Japan) was used for gene cloning of PCR. *P. pastoris* GS115 strain and pPIC9K vector purchased from Invitrogen (Carlsbad, CA, USA), and were used for laccase heterologous expression. Liquid medium (LM) (*w*/*v*) (2% poplar dust, 1% yeast extract, 0.2% KH_2_PO_4_, 0.2% (NH_4_)_2_SO_4_, 0.05% MgSO_4_·7H_2_O, 0.01% CaCl_2_) was used to induce laccase production in *T. hirsuta* MX2 as our previously report [8]. Minimal dextrose medium (MD) (*w*/*v*) (2% dextrose, 1.34% yeast nitrogen base, 4 × 10^−5^% biotin) was selected to screen recombinants of *P. pastoris*. Buffered glycerol-complex medium (BMGY) (*w*/*v*) (2% peptone, 1%, yeast extract, 1.34% yeast nitrogen base, 0.1 M potassium phosphate (pH 6.0), 0.5% glycerol, 4 × 10^−5^% biotin) was used to culture strain of *P. pastoris*. Buffered methanol-complex medium (BMMY) (*w*/*v*) (as BMGY, methanol instead of glycerol) was conducted to induce the production of recombinant laccase.

### 4.2. Cloning of Laccase Gene

MX2 mycelium grown in LM for seven days was harvested. Total RNA was isolated using MiniBest RNA Extraction kit (TaKaRa, Japan), and PrimeScript II cDNA Synthesis kit (TaKaRa, Japan) was used for synthesizing first strand cDNA according to the manufacturer’s instructions.

Based on laccase nucleotide sequence (*Lac1*) from *T. hirsuta* MX2 (GenBank accession number: MN327569), two primers of Lac1-F (5′-CGGAATTCGCCATTGGACCGAAGGCGAACCTCG-3′) and Lac1-R (5′-ATTTGCGGCCGCTCACAGATCGCCCTCCGCCAGCTTG-3′) were designed to amplify gene sequence encoding mature peptide of Lac1. Two restriction sites of *Eco*R I and *Not* I were introduced at the 5′ and 3′ ends of the PCR products, respectively. The PCR procedure was: initial denaturation at 98 °C for 10 s; 33 cycles of 98 °C for 10 s, 65 °C for 5 s, and 72 °C for 1.5 min; final extension at 72 °C for 10 min. The PCR products were analyzed on 1.5% agarose gel. The band of approximately 1.5 kb in size was purified, ligated into pMD 19-T and verified by sequencing.

To expression in *P. pastoris*, the sequence of *Lac1* was separated from recombinant pMD 19-T vector by *Eco*R I and *Not* I restriction enzymes, and was ligated into *Eco*R I-*Not* I-digested pPIC9K, designating pPIC9K-*Lac1*. The pPIC9K-*Lac1* was then linearized with *Sac* I, and was transferred into *P. pastoris* GS115 by electroporation. Positive transformants were selected after 3 days in MD medium.

### 4.3. Screening and Expression of Laccase Recombinants

In order to screen transformations with laccase activity, forty *P. pastoris* colonies were randomly picked up from the MD medium, and re-inoculated into a new MD plate. After 4 days of culture at 28 °C, the colonies were successively inoculated into a 96-well plate containing 500 µL BMMY medium, and were cultured at 28 °C for 24 h (250 rpm). ABTS (200 µL, 1 mM, pH 4.0) was added to BMMY medium and the colonies with laccase activity were detected by observing appearance of green color after a period of reaction. The greenest colony was selected for scale-up (1 L culture scale) production of laccase. Briefly, the colony was grown in 25 mL BMGY medium for 24 h at 28 °C (250 rpm). The cells were harvested by centrifugation at 4000 rpm for 5 min, resuspended in 1 L BMMY medium, and cultured at 28 °C and 250 rpm. During the colony culture, 10 mL methanol was added to BMMY medium daily to induce laccase production, and 1 mL culture liquid was collected every day for laccase activity determination.

### 4.4. Laccase Activity Assay

Laccase activity was determined as described in our previous report [8] by monitoring the absorbance at 420 nm for the oxidation of ABTS (Ɛ_420_ = 36,000 M^−1^ cm^−1^). For this, 0.5 mL of culture supernatant was mixed into 3 mL of 100 mM citrate phosphate buffer (pH 5.0) containing 0.5 mM ABTS and incubated at 25 °C. For purified rLac1, a 20 µL amount of enzyme was added to the 300 µL reaction mixture. One unit of laccase activity was defined as the amount of enzyme leading to the oxidation of 1 µmol of ABTS per min. Protein concentration was estimated using the Bradford method with bovine serum albumin as a standard.

### 4.5. Purification of Recombinant Laccase

Recombinant with high laccase activity was cultured in BMMY medium at 28 °C and shaken at 250 rpm. The culture supernatant was harvested at the day 5 by centrifugation (4000 rpm) at 4 °C, and filtered with a Millipore tube (cutoff 10 kDa proteins). The filtered supernatant was collected for (NH_4_)_2_SO_4_ precipitation (80% saturation), and the precipitated proteins were dialyzed with 20 mM citrate phosphate buffer (pH 5.0) at 4 °C for 12 h. The dialyzed sample was added to a pre-equilibrated DEAE-Sepharose FF column and separated by anion exchange chromatography. Eluted liquid contained 0.5 M NaCl in 20 mM citrate phosphate buffer (pH 5.0). The fractions with laccase activity were collected and concentrated by ultrafiltration. Molecular weight of rLac1 was determined using SDS-PAGE. Zymogram analysis was conducted using native-PAGE stained with ABTS.

### 4.6. Characterization of Recombinant Laccase

The optimal pH of rLac1 was estimated by determining enzyme activity toward ABTS in pH values of 2.0 to 6.0. The reactions were carried out at 30 °C in citrate phosphate buffer (100 mM). The pH stability was evaluated by calculating the residue activity after incubation rLac1 in buffer with pH values of 2.0 to 5.5 for 72 h. The optimal temperature was examined by measuring the enzyme activity of rLac1 between 0 and 90 °C. The thermostability of rLac1 was determined by evaluating the residual activity for 30 min in buffer with various temperatures (0–70 °C).

*K*_m_ and *k*_cat_ values of rLac1 against ABTS and DMP were determined at pH 5.0 and 50 °C using the Lineweaver–Burk double-reciprocal plot [8]. Laccase activity toward 2,6-dimethoxyphenol (DMP) was measured by recording the change of absorbance value at 469 nm (Ɛ_469_ = 27,500 M^−1^ cm^−1^).

The effect of metal ions (Mg^2+^, Cu^2+^, Mn^2+^, Zn^2+^, Na^+^, and Fe^2+^) and organic solvents (acetone, acetonitrile, dimethylsulfoxide, ethanol, methanol, and isopropanol) on the activity of rLac1 were investigated by measuring the relative activity of the enzyme to ABTS. Considering the stability and practical application of rLac1, the treatment conditions were set at pH 5.0 and 30 °C. The activity of rLac1 without any of the effectors was taken as 100%.

### 4.7. Dye Decolorization

The decolorization abilities of rLac1 to RBBR, AR1, CV, and NR were determined as described in Huang et al. [8]. 0.05 U/mL of rLac1 was mixed with 150 mg/L RBBR (λ_max_ = 592 nm), 15 mg/L AR1 (λ_max_ = 529 nm), 60 mg/LCV (λ_max_ = 590 nm), and 5 mg/LNR (λ_max_ = 530 nm) into 1 mL of reaction system, respectively. The decolorization reaction was performed at 28 °C in the absence or presence of ABTS (0.1 mM) in pH 5.0 buffer. The decolorization rate of each dye was calculated by measuring the decrease of the maximum absorbance of each dye after 3 h of reaction. The equation was as follows:

Decolorization rate (%) = [(Ai − Af)/Ai] × 100%(1)

here, A_i_ and A_f_ represented the initial and final absorbance of the reaction, respectively.

### 4.8. Molecular Docking between Laccase and Dyes

Molecular dockings between laccase and dye molecules were operated with the AutoDock tools [49]. The 3D structure of laccase (PDB: 1GYC) was retrieved from PDB (https://www.pdbus.org, accessed on 28 December 2021), and the original ligands and water molecules were removed from the laccase protein using PyMOL [50]. The 3D models of RBBR, AR1, CV, and NR were obtained from PubChem (https://pubchem.ncbi.nlm.nih.gov, accessed on 26 December 2021). After adding hydrogen, fixing charge and torsion, laccase protein and dye molecules were used for docking process. The grid box was arranged around the protein’s substrate binding cavity (SBC) [48]. The docking was accomplished with Lamarckian genetic algorithm [51]. Total ten bound conformations of each dye were created, and conformation with the best binding energy is used to visually understand the interaction between protein and dye molecules.

## 5. Conclusions

A laccase B gene from *T. hirsuta* MX2 was cloned and expressed in *P. pastoris*. A yeast recombinant with a yield of 2.59 U/mL of laccase was screened by methanol induction. The recombinant laccase was purified from culture medium and characterized. rLac1 showed the highest activity and pH stability under acidic condition. High concentration of Cu^2+^ (100 mM) is able to increase the activity of rLac1. It also displayed a certain tolerance to organic solvents. Additionally, rLac1 showed excellent decolorization ability to RBBR, and its decolorization rates of synthetic dyes were improved compared with the native laccase (Lac1). The recombinant laccase rLac1 seems to be activity-induced by copper and have the ability to enhancement dye decolorization.The results suggestedrLac1 has potential application prospect in textile wastewater removal of dyes.

## Figures and Tables

**Table 1:** Purification of rLac1 from *T. hirsuta* MX2.

Purification Step	Total Volume (mL)	Total Activity (U)	Total Protein (mg)	Specific Activity (U/mg)	Percent Recovery (%)	Purification Fold
Culture supernatant	870	2235	116.8	19.1	100	1
Ultrafiltrate	186	1971	53.6	36.8	88.2	1.9
(NH_4_)_2_SO_4_ precipitation	30	484	7.8	62.1	21.7	3.3
DEAE-Sepharose FF	14	129	1.4	92.1	5.8	4.8

**Table 2:** Kinetic constants of native (Lac1) and recombinant laccase (rLac1) from *T. hirsuta* MX2.

Substrate	Lac1 *			rLac1		
	*K*_m_ (µM)	*k*_cat_ (s^−1^)	*k*_cat_/*K*_m_ (M^−1^ s^−1^)	*K*_m_ (µM)	*k*_cat_ (s^−1^)	*k*_cat_/*K*_m_ (M^−1^ s^−1^)
ABTS	22.4	91.7	4.1 × 10^6^	28.4	343.2	1.2 × 10^7^
DMP	351.7	28.4	8.1 × 10^4^	394.1	141.7	3.6 × 10^5^

* Results were described by Huang et al. [8].

**Table 3:** Effect of organic solvents on the activity of rLac1.

Organic Solvents (*v*/*v*)	Relative Activity		
	5%	10%	50%
Acetone	69.1 ± 7.8	44.2 ± 3.9	0.4 ± 0.6
Acetonitrile	80.0 ± 12.3	61.3 ± 3.1	9.0 ± 0.2
Dimethylsulfoxide	64.5 ± 6.4	43.9 ± 1.4	4.3 ± 2.3
Ethanol	70.0 ± 4.3	53.3 ± 2.8	4.0 ± 1.0
Methanol	86.2 ± 7.3	62.4 ± 3.1	7.0 ± 0.9
Isopropanol	65.2 ± 9.8	42.5 ± 8.8	3.0 ± 0.9

**Table 4:** Dye decolorization of the recombinant (rLac1) *T. hirsuta* MX2 laccase.

Dyes	Mediator	Decolorization Rate (%)
RBBR	−	92.6 ± 0.9
	+	99.2 ± 0.6
AR 1	−	15.3 ± 0.8
	+	67.1 ± 0.4
CV	−	14.2 ± 0.4
	+	38.9 ± 0.9
NR	−	12.3 ± 0.3
	+	52.3 ± 0.5

− Decolorization by laccase in the absence of ABTS mediator. + Decolorization by laccase in the presence of ABTS mediator.

## Data Availability

The nucleotide sequence used to clone laccase gene is available from GenBank accession number MN327569. The crystal structure of laccase used for molecular docking is derived from PDB (ID: 1GYC).
